# Asphaltenes as Tackifiers for Hot-Melt Adhesives Based on Polyamide-12

**DOI:** 10.3390/polym18212637

**Published:** 2026-10-24

**Authors:** Viktoriya Y. Melekhina, Anna V. Vlasova, Sergey O. Ilyin

**Affiliations:** https://ror.org/01jgbgc06 A.V. Topchiev Institute of Petrochemical Synthesis, https://ror.org/05qrfxd25 Russian Academy of Sciences, 29 Leninsky Prospect, 119991 Moscow, Russia

**Keywords:** polyamide, asphaltenes, adhesives, hot-melts, rheology, adhesion strength, thermophysical properties

## Abstract

The potential of petroleum asphaltenes as novel, low-concentration tackifying agents in polyamide-12-based hot-melt adhesives was systematically investigated. The structural, rheological, thermophysical, mechanical, and adhesive properties of polyamide blends containing 5 to 20 wt% asphaltenes were comprehensively evaluated. A maximum twofold increase in lap-shear adhesion to steel was achieved at an optimal loading of 5 wt%, with the bond strength rising from 3.2 MPa to 6.9 MPa. This enhancement is attributed to the surfactant-like nature of asphaltenes. The peripheral aliphatic moieties of asphaltenes enable their partial dissolution within the polyamide matrix, enhancing interfacial affinity with the steel substrate without requiring the high loadings typical of conventional resin tackifiers. Mechanically, the tensile strength of the blends remained comparable to neat polyamide up to 10 wt%, whereas at 20 wt%, undissolved asphaltene aggregates acted as stress concentrators, reducing the cohesive strength from 42 MPa to 27 MPa. Rheologically, while the zero-shear viscosity increased only moderately (by a factor of 1.1–1.5), the absence of yield stress and a pronounced viscosity decrease at high shear stresses indicated excellent processability. Furthermore, the incorporation of asphaltenes caused no systematic changes in melting or crystallization temperatures, with the relative degree of crystallinity decreasing by only 0.3–6.4%. Ultimately, this work demonstrates that asphaltenes serve as highly efficient, sustainable tackifiers for polyamide hot-melts, offering a novel pathway for the valorization of heavy crude oil by-products.

## 1. Introduction

A hot-melt adhesive is a solvent-free, specialized fusible composition based on a thermoplastic polymer. It is designed to form adhesive bonds between various materials, including metals, at relatively low temperatures and to maintain the formed bond after cooling [1,2]. Hot-melt adhesives have found wide application in various industries, particularly in the woodworking and furniture industries, electronics, and other fields [3,4]. There are a number of advantages of hot-melt adhesives, such as fast bonding, environmental friendliness, cost-effectiveness, reduced fire hazard, versatility, moisture resistance, long shelf life, as well as excellent strength and adhesion properties [5]. Hot-melt adhesives usually consist of a polymer base and a tackifier [6,7,8]. The most widely used polymer bases for these adhesives are ethylene-vinyl acetate copolymers [1,9,10], polyisoprene [11], polyisobutylene [12,13], polyethylene [14], polybutene [15], polyvinyl acetate [16], polylactide [17], polyesteramide [18], polyurethane [19], polyester/polyamide blends [20], and others. The polymer base constitutes more than half of the adhesive composition and is responsible for its strength, elasticity, viscosity, and adhesion. Resins are the second component of adhesives, constituting about 30 wt%. They include both natural (rosins, terpenes, and their derivatives) [21] and synthetic resins (petroleum-based, terpene–phenolic, and indene–coumarone) [22,23,24,25]. Resins are used to enhance adhesive bonding, improve the wetting of the surfaces being joined, and reduce viscosity [26]. Additionally, hot-melt adhesives contain various additives (around 20 wt%), such as waxes [1,27], plasticizers [28], colorants, fillers, antioxidants [29], and others. Waxes are primarily added to slowly crystallizing polymer bases to shorten the open time and accelerate setting upon cooling, while also reducing melt viscosity, improving substrate wetting, and lowering formulation costs. Conversely, plasticizers are often introduced into fast-crystallizing or highly rigid polymer formulations to improve processability, enhance operational elasticity, and reduce overall material costs. Plasticizers facilitate the dispersion of ingredients, lower the molding temperature of the compositions, and improve the low-temperature resistance of polymers, although they may sometimes reduce their heat resistance. Certain plasticizers can also enhance the flame, light, and thermal stability of polymers [30]. The most common plasticizers are esters, such as dioctyl phthalate, dibutyl phthalate, dioctyl adipate, dioctyl sebacate, trioctyl phosphate, and trimellitates [31,32]. Chlorinated paraffins, mineral and vegetable oils, epoxidized soybean oil, and other liquids are also used [33].

Aliphatic polyamides (nylons) are specialized, fast-crystallizing, and rigid polymers with outstanding properties dictated by their specific monomeric composition [34]. These materials exhibit remarkable characteristics: they possess high strength, are resistant to a wide range of chemicals, withstand temperatures of up to 140 °C and higher, and provide excellent thermal and electrical insulation. Moreover, polyamides are highly compatible with a wide variety of fillers, which opens up endless possibilities for developing composite materials and modified plastics with precisely tailored properties [35].

The use of aliphatic polyamides as hot-melt adhesives was first reported in 1959 [36]. Due to their elevated softening points and excellent bond strength, polyamides are widely utilized in this field [37]. For instance, Chun et al. demonstrated that a terpolymer of nylon 6, nylon 66, and nylon 12, or a blend of nylons rather than a nylon homopolymer, serves as a suitable base resin for hot-melt adhesives, since the disruption of polymer chain regularity reduces crystallinity, leading to a lower melting point and melt viscosity [38]. Furthermore, the rheological properties of polyamide-based hot-melt adhesives can be controlled by incorporating terpene resins, butylbenzyl phthalate, and paraffin wax. Zhao et al. investigated hot-melt adhesives prepared by blending nylon 12 and bamboo flour at a weight ratio of 75/25, followed by selective laser sintering, demonstrating that their mechanical properties increased with the decrease in layer thickness [39]. Chen et al. studied the influence of the intrinsic viscosity of polyesteramides on their rheological, thermophysical, mechanical, and adhesive properties [18]. The results showed that the melt viscosity, tensile strength, elongation at break, and lap shear strength of the polyesteramides rose with increasing intrinsic viscosity, whereas their softening temperature, glass transition temperature, and hardness remained virtually unchanged as the intrinsic viscosity increased within a certain range.

A modern approach to polymer modification involves the increasing use of natural and industrial waste, notably coal- or petroleum-based asphaltenes [40]. Petroleum asphaltenes are constituents of heavy crude oil [41], contributing to its high viscosity, and remain as a residue after deasphalting, a process carried out to facilitate pipeline transportation [42,43]. The rational utilization of residual asphaltenes is a significant environmental challenge, which is being addressed by employing asphaltenes in the production of emulsions [44,45], carbon fibers [46,47], and bitumen binders [48,49]. Furthermore, asphaltenes serve as inexpensive fillers that enhance the performance properties and reduce the cost of various polymer materials, including epoxy plastics [50,51,52,53], polyethylene [54,55,56,57], polypropylene [58], polyisobutylene [59], polymethyl methacrylate [60], polystyrene [61,62], and styrene copolymers [63,64,65]. Asphaltene-modified plastics can be used in many industries, with the exception of applications involving direct contact with living organisms or food, due to the potential carcinogenicity of asphaltenes [66]. Moreover, asphaltenes act as surfactants that exhibit limited solubility in the polymer matrix while enhancing the tackiness of the polymer. Thereby, asphaltenes can be used as adhesion promoters for hot-melt adhesives [64,65]. However, despite the wide range of polymers previously modified with asphaltenes, asphaltene-containing polyamide hot-melt adhesives have not yet been developed and investigated.

The novelty of this study lies in exploring asphaltenes as a highly efficient, low-concentration alternative to traditional tackifying resins. Unlike conventional modifiers, which typically require high loadings to improve wetting and tack, we propose that asphaltenes can function as highly effective tackifiers for polyamides at minimal concentrations. Driven by the structural compatibility between the aliphatic moieties of asphaltenes and the aliphatic polyamide matrix, these additives are expected to enhance interfacial affinity at low loadings. This approach potentially maximizes adhesion without significantly compromising the polymer’s crystallinity or inducing a yield stress that would restrict melt flowability.

Among aliphatic polyamides, polyamide 12 (polylaurolactam, nylon 12) stands out due to its superior resistance to moisture and harsh chemical environments. Consequently, it is widely utilized in the manufacturing of insulating materials, as well as housing, sealing, and technical components for the automotive [67], shipbuilding [68], and mechanical engineering industries [69]. Driven by these robust properties, this study utilizes polyamide 12 as the base polymer matrix. The objective is to develop and investigate asphaltene-modified polyamide 12 formulations for use as advanced hot-melt adhesives. Specifically, the critical gap this research addresses is the complete absence of data on the interaction between asphaltenes and fast-crystallizing aliphatic polyamides in hot-melt systems. The originality of this study lies in the first experimental validation of asphaltenes as highly efficient, low-concentration tackifiers that operate via a surfactant-like mechanism, fundamentally differing from the high-loading, viscosity-reducing approach of traditional resins. The overall significance of this work extends beyond formulating a new adhesive, as it provides both technological and environmental benefits by creating a high-performance material while simultaneously establishing a novel, value-added pathway for the rational utilization of heavy crude oil by-products.

## 2. Materials and Methods

### 2.1. Materials

Polyamide 12 (PA12) produced by Anid (Yekaterinburg, Russia) with a melting point of 178 °C was used as a polymer base. Its intrinsic viscosity in m-cresol at 25 °C was 1.221 dL/g [70], corresponding to a weight-average molecular weight of 3.47 × 10^4^ g/mol [71].

The asphaltenes were isolated from heavy crude oil from the Ashalchinskoye oilfield (η_20 °C_ = 4.25 Pa·s, Tatarstan, Russia). According to the definition of the American Petroleum Institute, the oil density was 15.1 °API, and there was no evidence of crystallized paraffin wax. The SARA composition of the crude oil is 23.1% saturates, 45.6% aromatics, 23.8% resins, and 7.5% heptane-insoluble asphaltenes. The composition and rheological properties of the heavy crude oil were investigated in detail previously [41,43,72]. Hexamethyldisiloxane (EKOS-1, Moscow, Russia) was used as a precipitant at a volume ratio of precipitant to crude oil of 15:1. The asphaltene isolation procedure has been described in detail previously [73]. The average molecular weight of the asphaltenes was 828 g/mol, and their elemental composition was C/H/N/S = 82.3/9.1/1.6/5.8 wt%.

### 2.2. Blend Preparation

The asphaltene-containing polyamide blends were prepared by melt mixing using a PolyDrive twin-rotor laboratory mixer (Haake, Vreden, Germany) equipped with sigma rotors. The polyamide and asphaltenes were mixed at weight ratios of 95/5, 90/10, and 80/20 for 1 h at 180 °C and a rotational speed of 30 rpm.

Films from asphaltene/polyamide blends were formed using an HLCL-1000 laminator (Cheminstruments, West Chester Township, OH, USA) between two layers of a siliconized polyimide-based antiadhesive film at 180 °C to evaluate the strength properties and the structure of asphaltenes in polyamide. The thickness of the prepared films was 330 ± 20 μm.

Figure 1 schematically illustrates the overall workflow of the study, encompassing blend preparation and the comprehensive characterization of the resulting properties.

### 2.3. Methods

The morphology of thin films was evaluated from microphotographs obtained using an MBI-1 microscope (LOMO, Saint Petersburg, Russia) equipped with a 40× objective lens and an IMX226 digital camera (Sony, Tokyo, Japan) with a 12 MP resolution and a 1/1.7-inch sensor size.

X-ray diffraction (XRD) patterns of the asphaltene/polyamide films were recorded using a Rotaflex RU-200 diffractometer (Rigaku, Tokyo, Japan) equipped with a rotating copper anode and a D/Max-B horizontal wide-angle goniometer (Rigaku). The measurement range of the diffraction angles (2*θ*) was 3° to 90°. The measurements were performed in continuous scanning mode at a rate of 4 °C·min^−1^ and a step of 0.04°. The wavelength of the monochromatic radiation was 1.5405 Å.

Differential scanning calorimetry (DSC) was used to evaluate the effect of asphaltenes on the melting and crystallization temperatures of polyamide. The mixtures were investigated using an MDSC 2920 calorimeter (TA Instruments, New Castle, DE, USA) under an argon atmosphere. The samples were heated from room temperature to 200 °C at a rate of 10 °C·min^−1^, followed by cooling at the same rate.

The rheological properties of the blend melts were investigated at 180 °C using a DHR-2 rotational rheometer (TA Instruments, New Castle, DE, USA) equipped with a cone-and-plate geometry (with a plate diameter of 25 mm and a cone angle of 2°). The dependencies of the viscosity (*η*) on the shear stress (*σ*) were obtained by stepwise increasing the shear rate from 0.001 to 100 s^−1^. The amplitude dependencies of the storage (*G*′) and loss (*G*″) moduli were measured at an angular frequency (*ω*) of 6.28 rad·s^−1^ through a stepwise increase in the strain amplitude (*γ*) from 0.01 to 1000%. The frequency dependencies of the storage and loss moduli were obtained in the linear viscoelastic region of the samples by varying the angular frequency from 0.0628 to 628 rad·s^−1^. To ensure data reliability, each rheological measurement was independently repeated 2–3 times. The presented curves are representative, as the replicate measurements demonstrated high reproducibility with minimal variability between runs.

Tensile strength, Young’s modulus, and elongation at break were measured using dumbbell-shaped specimens die-cut from the asphaltene/polyamide films. The specimens featured a narrow parallel section width of 3 mm and a gauge length of 25 mm. The measurements were performed on a TT-1100 tensile testing machine (Cheminstruments, Fairfield, OH, USA) at 25 °C and a stretching rate of 3.8 cm/min. A minimum of seven replicate specimens were tested for each formulation, and the reported values represent the average.

For lap shear adhesion testing, two galvanized steel plates with a width of 10 mm and a thickness of 250 μm were bonded in a single-lap joint configuration using the test adhesive at 200 °C, forming an overlap area of 10 × 5 mm. The plates were pressed together under a constant load of 1 kgf for 1 min, cooled to room temperature, and subsequently unloaded. The lap shear adhesion strength was evaluated on an I1140M tensile testing machine (Tochpribor-KB LLC, Ivanovo, Russia) at a crosshead speed of 10 mm/min and 25 °C. At least five replicate joints were evaluated for each adhesive formulation, with average values reported.

Statistical analysis of the mechanical and adhesive properties was performed using Welch’s *t*-test (a two-sample Student’s *t*-test assuming unequal variances) to compare the asphaltene-modified blends with the neat polyamide-12 control.

## 3. Results and Discussion

### 3.1. Structure

The particle size and the presence of asphaltene aggregates in the modified polyamide were evaluated using transmission optical microscopy. Asphaltene-filled polyamide blends were used in the form of thin films placed between two cover glasses. When asphaltenes were added to the colorless polyamide, the blend samples acquired a yellow-brown coloration in a thin layer (Figure 2). This coloration suggests at least partial dissolution of the asphaltenes within the polymer matrix, since complete insolubility would have resulted in a colorless matrix interspersed with distinct, dark particles. The undissolved asphaltene domains observed in the polyamide are restricted to the micrometer scale, demonstrating a high degree of dispersion. Notably, no large-scale asphaltene aggregation was observed, even at high filler loadings of up to 20 wt%.

Thus, utilizing an aliphatic polymer matrix, which structurally complements the peripheral aliphatic moieties of asphaltenes [74,75], fosters strong polymer–asphaltene interactions. This inherent compatibility consequently ensures high filler dispersibility and effectively prevents the formation of aggregates within the polymer matrix.

According to the diffractograms (Figure 3), the neat polyamide is in a semicrystalline state, with characteristic reflections observed at 2*θ* values of 5.5°, 11.1°, and 21.3°, which correspond to the (020), (040), and (001) crystallographic planes, respectively, of the monoclinic γ-form of PA12 prepared by melt cooling [76,77,78]. The incorporation of asphaltenes leads to a noticeable decrease in the intensity of these diffraction peaks, which may be attributed to a reduction in overall crystallinity, an increase in crystal defects, a decrease in crystallite size, or changes in preferential crystal orientation relative to the sample surface [79]. At the same time, the main diffraction peak shifts from 21.3° for neat PA12 to 20.6° for the composite containing 20 wt% asphaltenes. According to Bragg’s law, this corresponds to a slight increase in the interplanar *d*-spacing from 0.417 nm to 0.431 nm, indicating that the presence of asphaltenes expands the polymer chain packing within the crystalline domains.

### 3.2. Thermophysical Properties

The addition of asphaltenes has a negligible effect on the melting behavior of the polyamide. In the case of the blend containing 5 wt% of asphaltenes, the endothermic peak shifts slightly to a higher-temperature region, while higher asphaltene concentrations have practically no effect on the melting temperature (Figure 4a). In other words, asphaltenes neither reduce the polyamide crystal size nor increase crystal defects. The melting temperatures (*T*_m_) and enthalpies (Δ*H*_m_) of the blends are presented in Table 1.

The crystallization temperature of the polyamide (*T*_cr_) remains practically unchanged upon the addition of asphaltenes (Figure 4b), although the overall crystallization enthalpy of the blends (Δ*H*_cr_) decreases slightly. Thus, it is unlikely that asphaltenes act as nucleation sites, since otherwise the crystallization temperature would have increased.

To quantitatively assess the impact of asphaltene incorporation on the crystallinity of the polyamide matrix, the relative degree of crystallinity (*RDC*) was calculated as follows [80]:

(1) $$RDC=\frac{\left(\Delta H_{m}+\Delta H_{cr}\right)}{\left(1-w_{asph}/100)(\Delta H_{m,PA}+\Delta H_{cr,PA}\right)}\cdot 100\%,$$

where *w*_asph_ is the asphaltene content (in wt%), while Δ*H*_m,PA_ and Δ*H*_cr,PA_ are the specific melting and crystallization enthalpies of the neat polyamide, respectively.

*RDC* calculation shows that the crystallinity degree of the polyamide decreases by 0.3–6.4% relative to its initial value (Table 1). In other words, asphaltenes have practically no effect on the polymer matrix crystallinity.

Finally, it should be noted that because both PA12 and petroleum asphaltenes exhibit high thermal stability with degradation onset temperatures well above 350 °C, thermal decomposition does not occur at typical hot-melt processing temperatures (180–200 °C). Given this substantial thermal margin, the thermophysical characterization focused exclusively on DSC and rheological measurements, which govern the critical melting, flow, and solidification behavior required for hot-melt applications.

### 3.3. Rheological Properties

The addition of asphaltenes increases the maximum Newtonian viscosity of polyamide by a factor of 1.1–1.5 (Figure 5). In this case, the most significant increase in viscosity is observed with the addition of 10–20 wt% of asphaltenes, when a portion of the asphaltenes forms a liquid dispersed phase (Figure 2b,c), since the test temperature exceeds the glass transition temperature of the asphaltenes (approximately 63 °C [64]). The increase in viscosity is likely due to interfacial viscoelasticity [81,82], specifically at the asphaltene–polyamide interface. Remarkably, at high shear rates and shear stresses, the viscosity of the composites is lower than that of the unfilled polymer matrix. Such behavior can be explained by the deformation of asphaltene droplets and interfacial slip at the polymer–asphaltene boundary due to their poor adhesion [83,84]. At the same time, even at high filler loadings, the flow curves of the samples are not characterized by a yield stress, which suggests the absence of an asphaltene percolation network [85,86].

The neat polyamide melt exhibits linear viscoelastic behavior up to a strain amplitude of about 50% (Figure 6), which is consistent with conventional polymer melts and solutions [87,88]. In this regime, the loss modulus (characterizing energy dissipation during deformation) exceeds the storage modulus (characterizing energy storage), indicating that the melt exhibits predominantly liquid-like behavior. The addition of asphaltenes does not significantly alter the storage modulus but reduces the loss modulus. Furthermore, this reduction in the loss modulus becomes more pronounced with increasing asphaltene content. Notably, the extent of the linear viscoelastic region remains largely unaffected by the filler addition.

The frequency dependencies of the storage and loss moduli of the neat polyamide are typical for linear polymers (Figure 7). An increase in angular frequency leads to growth in both moduli, with the storage modulus rising more rapidly than the loss modulus, in accordance with the Maxwell model [89]. The incorporation of asphaltenes induces a dual, frequency-dependent effect: it increases both dynamic moduli at low frequencies, but decreases them at high frequencies. Notably, at an asphaltene concentration of 10 wt%, the storage modulus exceeds the loss modulus at high frequencies, indicating a transition toward solid-like, rubbery behavior in the melt. The decrease in the moduli at high frequencies is likely due to the partial solubility of low-molecular-weight asphaltenes in the polymer. In this case, asphaltenes act as a plasticizer for the polyamide, reducing its viscosity and stiffness due to an increased distance between macromolecular entanglements. In contrast, at low frequencies, asphaltenes increase the dynamic moduli, with a more pronounced effect on the storage modulus, which may be attributed to the elasticity of the interfacial boundary of the emulsified asphaltenes.

Thus, asphaltenes increase the viscosity of the polyamide melt, especially at concentrations of 10–20 wt%. This behavior fundamentally contrasts with that of conventional hot-melt adhesives modified with traditional tackifiers (e.g., hydrocarbon resins or waxes), which typically require high loadings (20–40 wt%) specifically to plasticize the matrix and reduce melt viscosity. Nevertheless, from a practical application standpoint, this moderate increase in viscosity does not critically hinder the use of these blends as hot-melt adhesives for several reasons. First, the optimal adhesive performance is achieved at a low asphaltene loading of 5 wt%, where the viscosity increase is minimal. Second, the absence of yield stress ensures that the melt retains its ability to flow and wet substrates even under low applied stresses. Third, and most importantly, at the high shear rates typical of industrial application processes (e.g., extrusion or roller coating), the viscosity of the asphaltene-containing composites is actually lower than that of the neat polyamide due to interfacial slip, which actively facilitates flow during processing. Finally, any moderate increase in low-shear viscosity can be readily compensated by a slight elevation in the application temperature, a standard and easily adjustable parameter in industrial hot-melt processing equipment.

### 3.4. Strength and Adhesion Properties

The tensile strength (*τ*_C_) and elongation at break (***δ***) of the polyamide films remain comparable to those of neat PA12 at 5 and 10 wt% asphaltene loadings. However, at 20 wt%, both parameters decrease significantly (Table 2). This decline is attributed to the fact that a 20 wt% loading exceeds the solubility limit of asphaltenes in the PA12 matrix. The excess asphaltenes form a distinct, dispersed phase of undissolved aggregates (as observed in Figure 2c), which act as stress concentrators under mechanical load. These defects initiate microcracks and lead to premature cohesive failure of the bulk polymer. Regarding Young’s modulus (*E*), a modest 1.2- to 1.3-fold increase is observed upon the addition of asphaltenes, regardless of their concentration. In general, it can be concluded that blends containing up to 10 wt% asphaltenes are comparable in strength to the base polyamide, since their strength properties vary within the margin of measurement error.

The incorporation of asphaltenes into polyamide profoundly impacts the lap-shear adhesive strength of bonded joints. A maximum two-fold increase in adhesion is achieved at an optimal asphaltene loading of 5 wt%, reaching 6.9 MPa. At this concentration, the majority of the asphaltenes dissolve within the polymer matrix, likely enhancing interfacial affinity between the polyamide and the steel substrate through asphaltenes’ surfactant-like behavior [53,64]. This adhesive enhancement is maintained up to a 10 wt% asphaltene loading. However, a further increase in concentration to 20 wt% causes a sharp decline in lap-shear strength, dropping to 1.5 MPa. At this high loading, the adhesive joint presumably undergoes cohesive failure within the weakened, defect-rich polymer matrix. This bulk failure mechanism completely overrides any interfacial adhesion benefits provided by the dissolved asphaltene fraction.

Based on the structural, rheological, and adhesive data, the interaction mechanism between asphaltenes and the PA12 matrix can be described as a two-level process. First, at the bulk level, the peripheral aliphatic moieties of the asphaltene molecules exhibit structural compatibility with the aliphatic backbone of PA12. This fosters the partial solubilization of lighter, lower-molecular-weight asphaltene fractions within the amorphous regions of the polymer, as evidenced by the uniform coloration of the films, the absence of a yield stress, and the plasticizing effect observed at high frequencies. Second, at the interfacial level, asphaltenes exhibit surfactant-like behavior. Their amphiphilic nature allows the polar polyaromatic cores (rich in heteroatoms such as N and S) to interact favorably with the native metal oxide layer on the steel substrate, while their aliphatic chains remain entangled within the PA12 matrix. This dual affinity effectively bridges the polymer and the steel, enhancing interfacial adhesion without requiring high asphaltene loadings, which would otherwise compromise the cohesive strength of the polymer.

## 4. Conclusions

Asphaltenes serve as highly efficient tackifying modifiers for polyamide-12, significantly enhancing adhesive properties without compromising the polymer’s cohesive strength. At an optimal loading of 5 wt%, the lap-shear strength of PA12/steel joints doubles from 3.2 to 6.9 MPa, while the bulk tensile strength remains comparable to that of neat PA12 (about 42 MPa). This enhancement is effective up to 10 wt%, whereas at 20 wt%, undissolved asphaltene aggregates act as defects, degrading both the cohesive strength of the blend (down to 27 MPa) and the overall bond strength (down to 1.5 MPa). A key novelty of this approach, compared to traditional tackifiers, is that this performance is achieved at a fraction of the typical loading, and without the melt viscosity reduction upon which conventional resin-based tackification relies. Instead, the viscosity increases only moderately (by a factor of 1.1–1.5). This indicates that the adhesion enhancement is governed by the surfactant-like behavior of asphaltenes—specifically, their partial solubilization in the aliphatic matrix, which improves interfacial affinity with the steel substrate—rather than by melt dilution. Furthermore, asphaltene incorporation induces no systematic changes in the melting or crystallization temperatures, nor in the relative degree of crystallinity of PA12, which decreases by only 0.3–6.4%. Notably, no yield stress emerges in the melt, even at 20 wt% loading. Consequently, the fundamental structure and processing characteristics of the polyamide remain largely intact, as any moderate viscosity increase can be compensated, if necessary, by raising the application temperature. Practically, these asphaltene-modified polyamides are suitable as high-performance hot-melt adhesives, as well as insulating and protective coatings for steel substrates (sheets, rods, and cables) against mechanical abrasion and environmental degradation. Ultimately, this work presents a novel, value-added pathway for the rational utilization of asphaltenes, transforming a ubiquitous by-product of heavy crude oil processing into a high-performance polymeric modifier. Despite these promising findings, this study has certain limitations that should be addressed in future research. First, the current work focuses primarily on the initial adhesive and mechanical properties, without evaluating long-term durability factors such as thermal aging, ultraviolet stability, or performance under humid conditions. Second, adhesion was assessed exclusively on steel substrates under standard ambient conditions. Future research will aim to evaluate the long-term environmental stability of these adhesive joints, investigate bonding to a wider range of substrates, and provide direct molecular-level evidence of the asphaltene–steel interface using advanced surface-sensitive techniques.

## Figures and Tables

**Figure 1:**
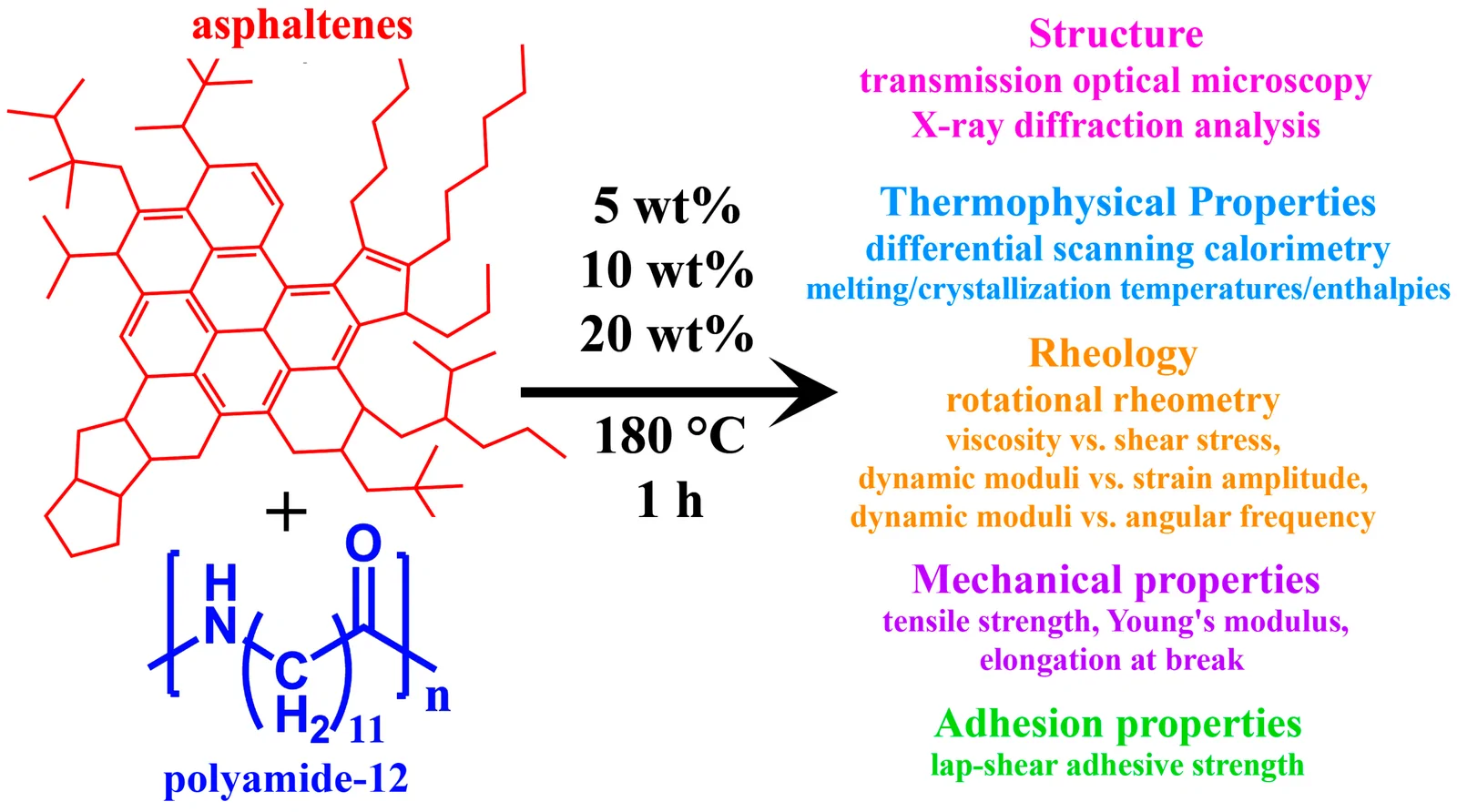
Schematic illustration of the experimental workflow: preparation of asphaltene/PA12 blends via melt mixing and the comprehensive characterization of their structural, thermophysical, rheological, mechanical, and adhesive properties.

**Figure 2:**
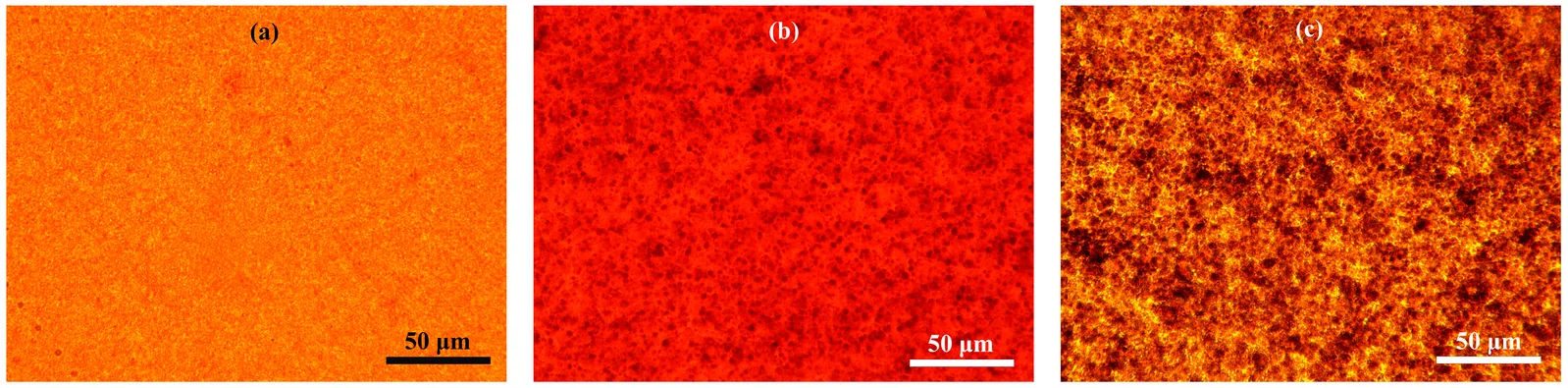
Microphotographs of polyamide films containing 5 (**a**), 10 (**b**), and 20 wt% (**c**) asphaltenes at 25 °C.

**Figure 3:**
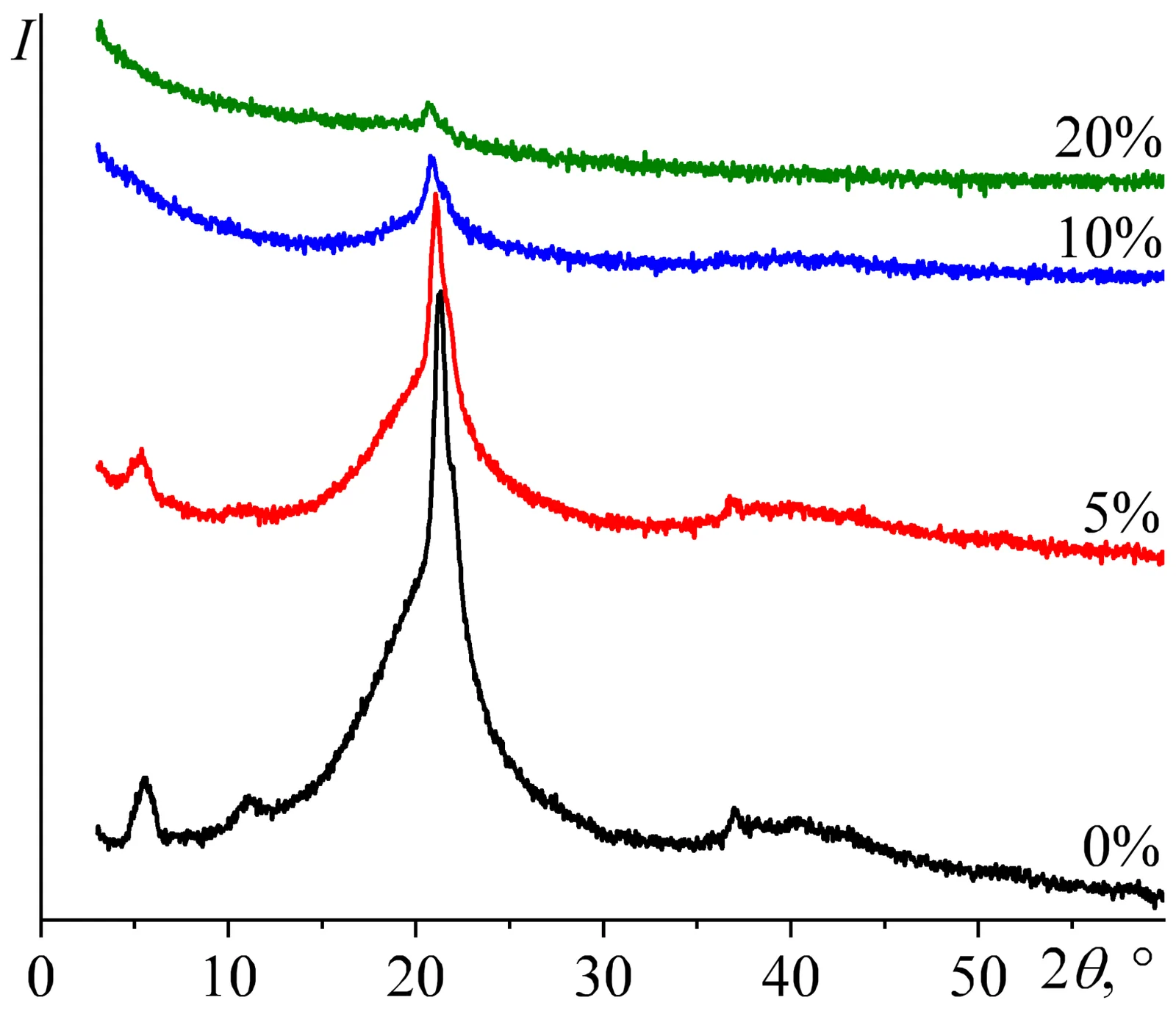
XRD patterns of polyamide films containing asphaltenes. The asphaltene mass content is indicated near the curves.

**Figure 4:**
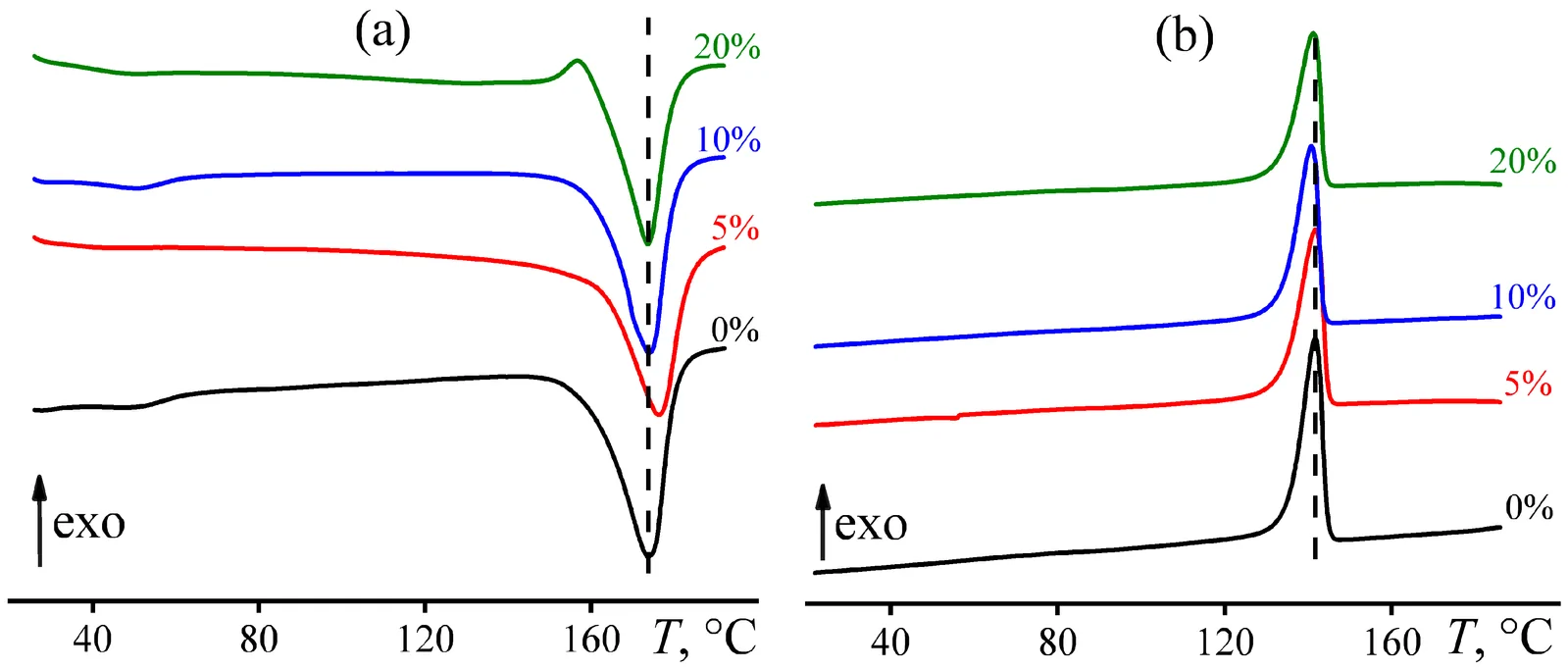
DSC thermograms of polyamide and its blends with asphaltenes in heating (**a**) and cooling (**b**) modes. The asphaltene mass content is indicated near the curves.

**Figure 5:**
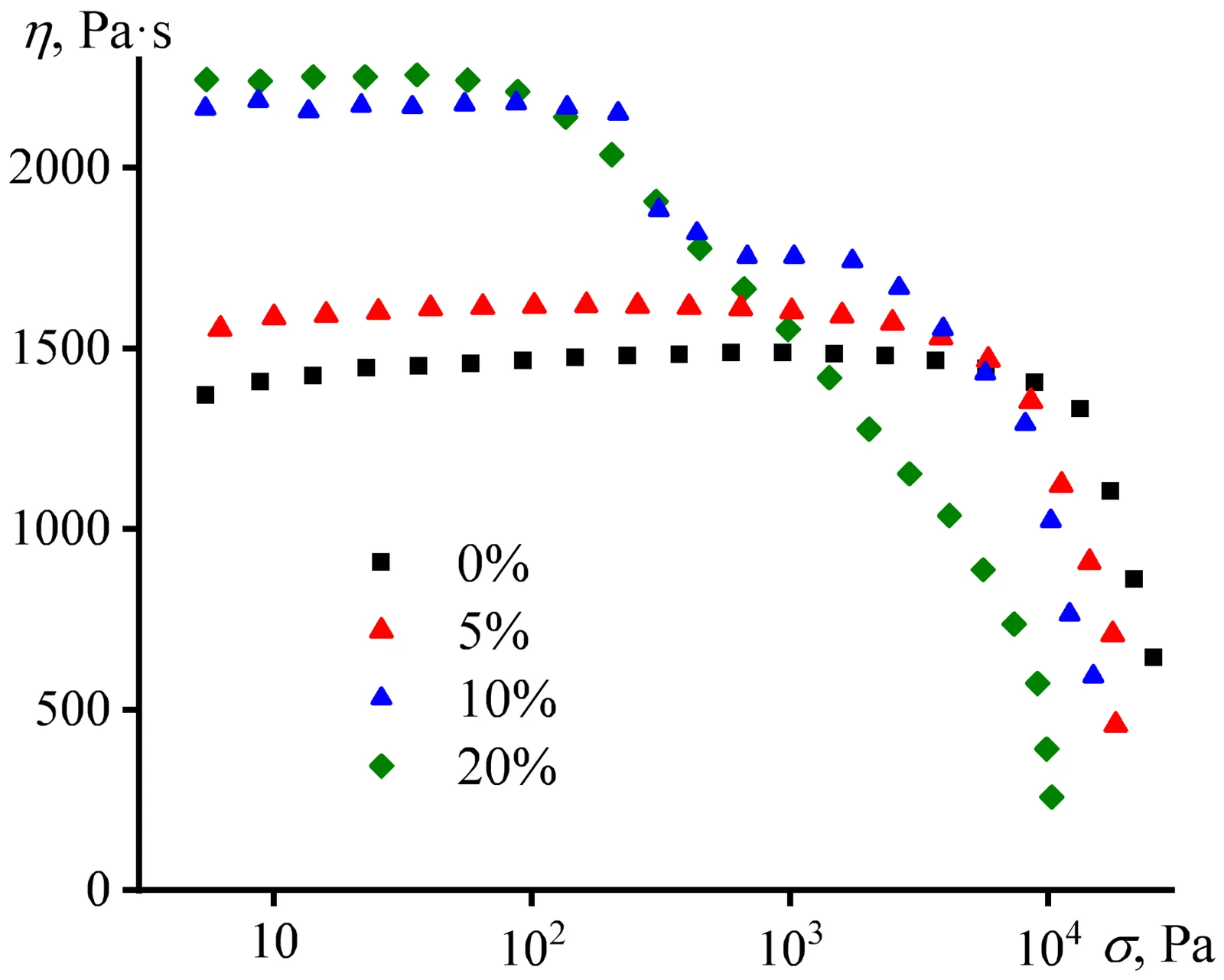
Dependencies of viscosity on shear stress for asphaltene/polyamide blends at 180 °C. The asphaltene mass content is indicated in the legend.

**Figure 6:**
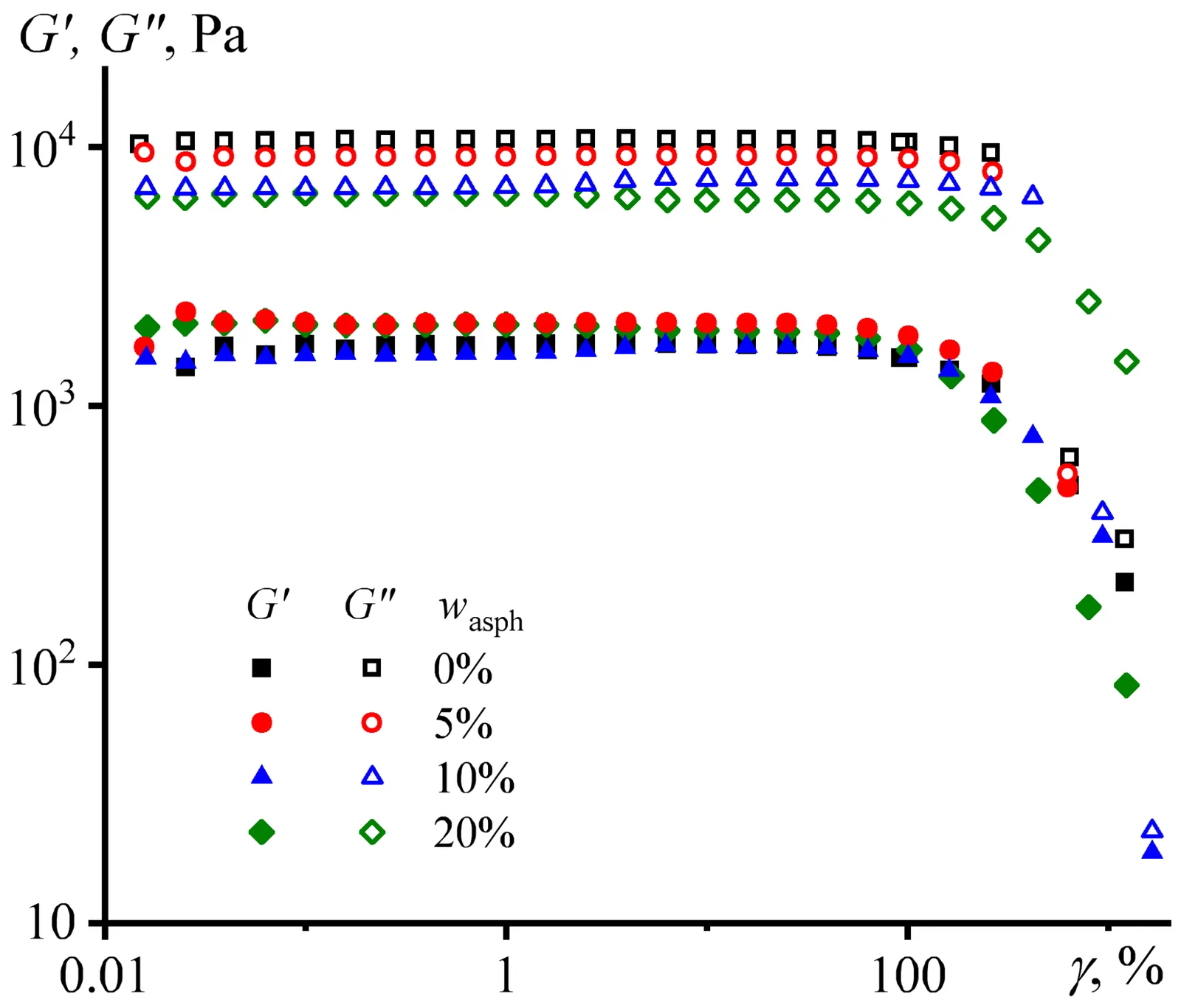
Amplitude dependencies of storage and loss moduli for asphaltene/polyamide blends at 180 °C and an angular frequency of 6.28 rad/s. The asphaltene mass content is indicated in the legend.

**Figure 7:**
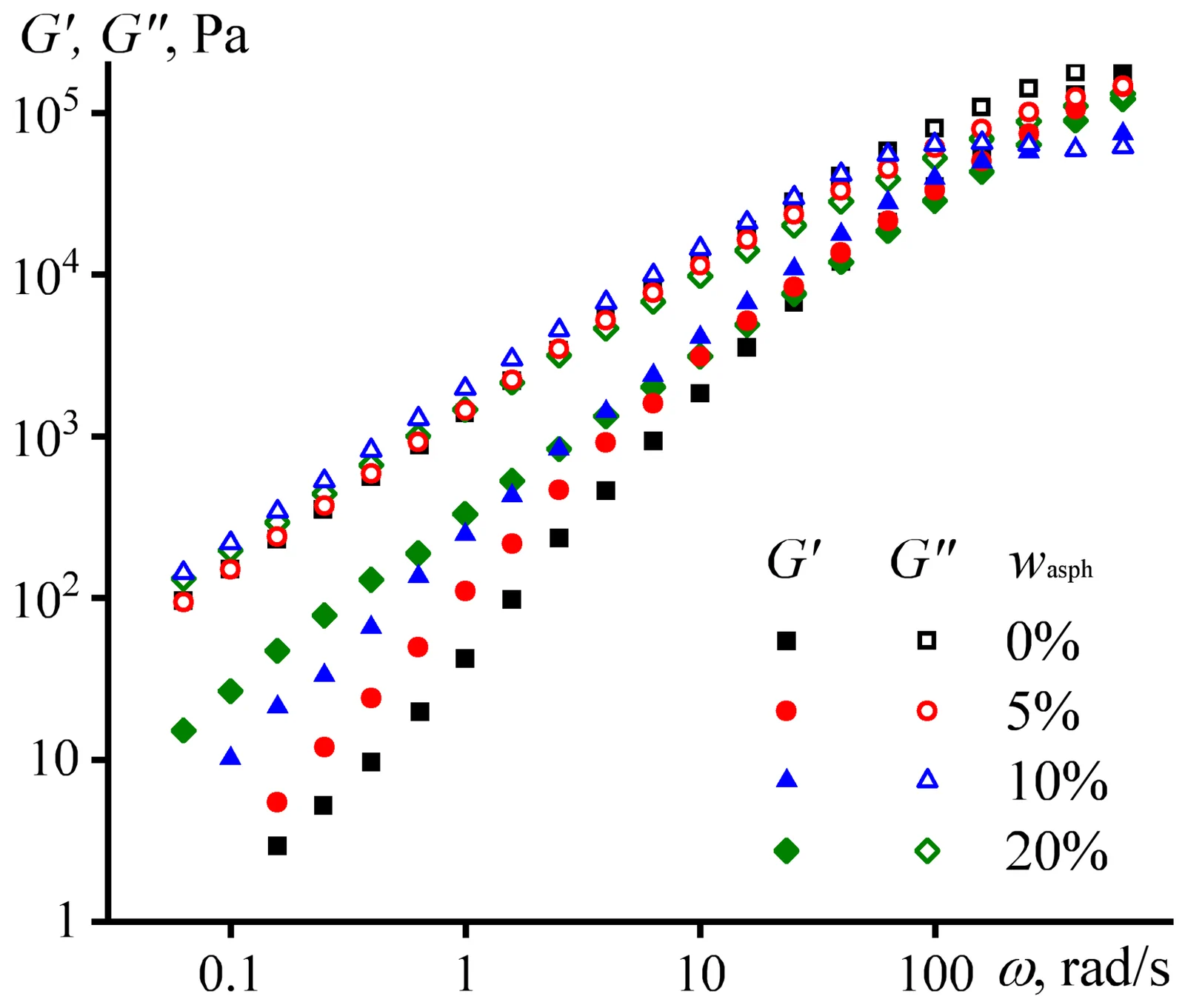
Frequency dependence of storage and loss moduli for asphaltene/polyamide blends at 180 °C. The asphaltene mass content is indicated in the legend.

**Table 1:** Melting and crystallization of asphaltene/polyamide blends.

*w*_asph_, wt%	*T*_m_, °C	Δ*H*_m_, J/g	*T*_cr_, °C	Δ*H*_cr_, J/g	*RDC*, %
0	173.9	59.8	141.6	58.0	100
5	176.3	51.2	141.7	53.5	93.6
10	174.1	53.4	140.8	52.3	99.7
20	173.7	45.9	141.2	46.5	98.1

**Table 2:** Strength and adhesion properties of asphaltene/polyamide blends.

*w*_asph_, %	*τ_C_,* MPa	*δ,* %	*E*, MPa	*τ*_A_, MPa
0	42 ± 8	380 ± 90	290 ± 30	3.2 ± 0.9
5	39 ± 4 ^d^	270 ± 50 ^d^	380 ± 50 ^c^	6.9 ± 1.6 ^b^
10	43 ± 3 ^d^	370 ± 30 ^d^	350 ± 30 ^c^	5.2 ± 1.2 ^c^
20	27 ± 4 ^c^	130 ± 100 ^b^	350 ± 90 ^d^	1.5 ± 0.7 ^c^

Note: The letter after the experimental data shows the *p*-value (from Welch’s *t*-test)—the probability that asphaltenes actually affected this parameter [90]: ^a^ *p* < 0.01 (good evidence that it does); ^b^ 0.01 < *p* < 0.05 (moderate evidence); ^c^ 0.05 < *p* < 0.1 (weak evidence); ^d^ *p* > 0.1 (no evidence).

## Data Availability

The data presented in this study are available on request from the corresponding author.
